# Predicting the mean first passage time (MFPT) to reach any state for a passive dynamic walker with steady state variability

**DOI:** 10.1371/journal.pone.0207665

**Published:** 2018-11-29

**Authors:** Isuri Wijesundera, Malka N. Halgamuge, Ampalavanapillai Nirmalathas, Thrishantha Nanayakkara

**Affiliations:** 1 Department of Infrastructure Engineering, The University of Melbourne, Melbourne, Australia; 2 Department of Electrical and Electronic Engineering, The University of Melbourne, Melbourne, Australia; 3 Dyson School of Design Engineering, Imperial College London, London, United Kingdom; Cinvestav-Merida, MEXICO

## Abstract

Idealized passive dynamic walkers (PDW) exhibit limit cycle stability at steady state. Yet in reality, uncertainty in ground interaction forces result in variability in limit cycles even for a simple walker known as the Rimless Wheel (RW) on seemingly even slopes. This class of walkers is called metastable walkers in that they usually walk in a stable limit cycle, though guaranteed to eventually fail. Thus, control action is only needed if a failure state (i.e. RW stopping down the ramp) is imminent. Therefore, efficiency of estimating the time to reach a failure state is key to develop a minimal intervention controller to inject just enough energy to overcome a failure state when required. Current methods use what is known as a Mean First Passage Time (MFPT) from current state (rotary speed of RW at the most recent leg collision) to an arbitrary state deemed to be a failure in the future. The frequently used Markov chain based MFPT prediction requires an absorbing state, which in this case is a collision where the RW comes to a stop without an escape. Here, we propose a novel method to estimate an MFPT from current state to an arbitrary state which is not necessarily an absorbing state. This provides freedom to a controller to adaptively take action when deemed necessary. We demonstrate the proposed MFPT predictions in a minimal intervention controller for a RW. Our results show that the proposed method is useful in controllers for walkers showing up to 44.1% increase of time-to-fail compared to a PID based closed-loop controller.

## Introduction

A passive walker is a simple form of legged locomotion which can move down a slope without actuation [1]. Its only source of energy is gravity. The energetics of these walkers are such that the energy lost in friction and collisions are balanced by the conversion of potential energy to kinetic energy as the walker moves down a slope [2]. In a previous study we have shown that passive dynamic walkers have state space attractor’s that can be efficiently used to arrive at this energy equilibrium despite different initial conditions [3]. The dynamics of a passive walker, though seemingly deterministic, is in fact highly complex due to the inevitable variability of punctuated state transitions at every impact with the ground even under idealized conditions.

A simplest of passive dynamic walkers, known as the rimless wheel (RW) has been studied widely [4–7] to understand the coupled dynamics of the moving body and the ground. The authors in [8] discuss the steady step period of RW calculated solving the equations of boundary conditions. Experiments in [3] show variability across steps even after reaching the steady state is predominantly dependent on the distribution of the coefficient of restitution (*η*) and that of friction (*μ*) in the ground. In this paper we extensively refer to the dynamics of the RW to demonstrate the proposed methods.

Dynamic systems with remarkably long life cycles that are yet guaranteed to eventually fail are called meta-stable dynamic systems. These systems show variability in state transitions even in steady state and will reach an absorbing (failure) state with the probability of one as time tends to infinity [9]. This means that in steady state, while small perturbations do not affect the stability of the system, larger ones can move the system state out of the attraction zone of the meta-stable state and result in failure. These systems are neither stable nor unstable and usually take a long time to reach failure. Many passive or minimally actuated dynamic walking systems fall under this category showing impressively long periods of continuous walking most of the time. The authors in [10] have shown that walking is well characterized as meta-stable processes, and that the stochastic dynamics of walking should be accounted for during control design in order to improve stability.

When concerned with designing dynamic walkers, a common goal is to maximize the expected time-to-failure [9–11]; or in other words, to increase the time before the walking comes to a stop. There are two main approaches of addressing this issue. The first is to evaluate the stability of the system for bounded inputs [10]. The second approach is to use real time adaptive control in order to delay a meta-stable system from reaching failure states. This can be done either by controlling in the state space in the form of minimal actuation [12, 13] or by making an indirect influence on the attractors of states through adaptation of internal impedance of the walker using prior knowledge [14]. Designs of actuation-based controllers for otherwise passive walkers usually have two main objectives: Increase lifespan of the system and achieve maximum energy efficiency. Controlling an otherwise passive walker with actuation in order to increase its stable lifespan is a complicated problem. Incorrectly applied external force will not only under-utilize energy but also might force the system to instability. Research has been done to develop methods that use the attraction fields around the steady state to optimize the control action where conditions leading to reaching the goal will stabilize the goal [14, 15].

Methods that take a Markov chain approach using an eigenmode analysis on the state transition probability matrices (TPM) [9, 16] are limited to providing a global value for reaching the absorbing state (failure state). A global mean first passage time (MFPT) prediction is a valuable tool in optimizing stability through system parameters. In contrast, a method which is able to predict time of arrival at ‘any’ state, including a failure state, is a better candidate as a feedback signal for a closed-loop controller of a passive dynamic robotic walker to make behavioural corrections only when needed. In other words, if we can develop a method to calculate the MFPT to reach any pre-defined target state (*T*), which is the time taken for the state vector to first reach *T* starting from any given source point (*S*), this could be used as a feedback signal.

In this paper we present a novel model to predict the mean time for a PDW system to reach any arbitrary state. The proposed method is an extension of a powerful technique proposed in [17], and further allows estimating MFPT to reach an arbitrary state from any given state even if the state transition probability distribution is biased in the state space. This presents a very useful tool in analyzing the stability of a passive walker because the state space variability of a PDW system follows the motion of a random walk biased by the state space attractors of meta-stable and failure states. Here, we model the system state of a passive walker on the return map space of its angular velocity at the collision and the state transitions for the walker are modelled as anisotropic random walks in this state space. In results, we compare the proposed MFPT prediction method with a Markov chain based method and show a 10% higher prediction accuracy with experimental data of the RW. We then follow by presenting a minimal-intervention controller for a RW where the MFPT predictions to reach identified states are used as feedback signals in a closed-loop controller to apply external torque to economically postpone failure. Finally, we compare the controller’s performance with that of a PID (proportional-integral-derivative) based controller to demonstrate the higher effectiveness of the proposed method.

## Materials and methods

### Passive dynamic motion of a RW

**Fig 1:**
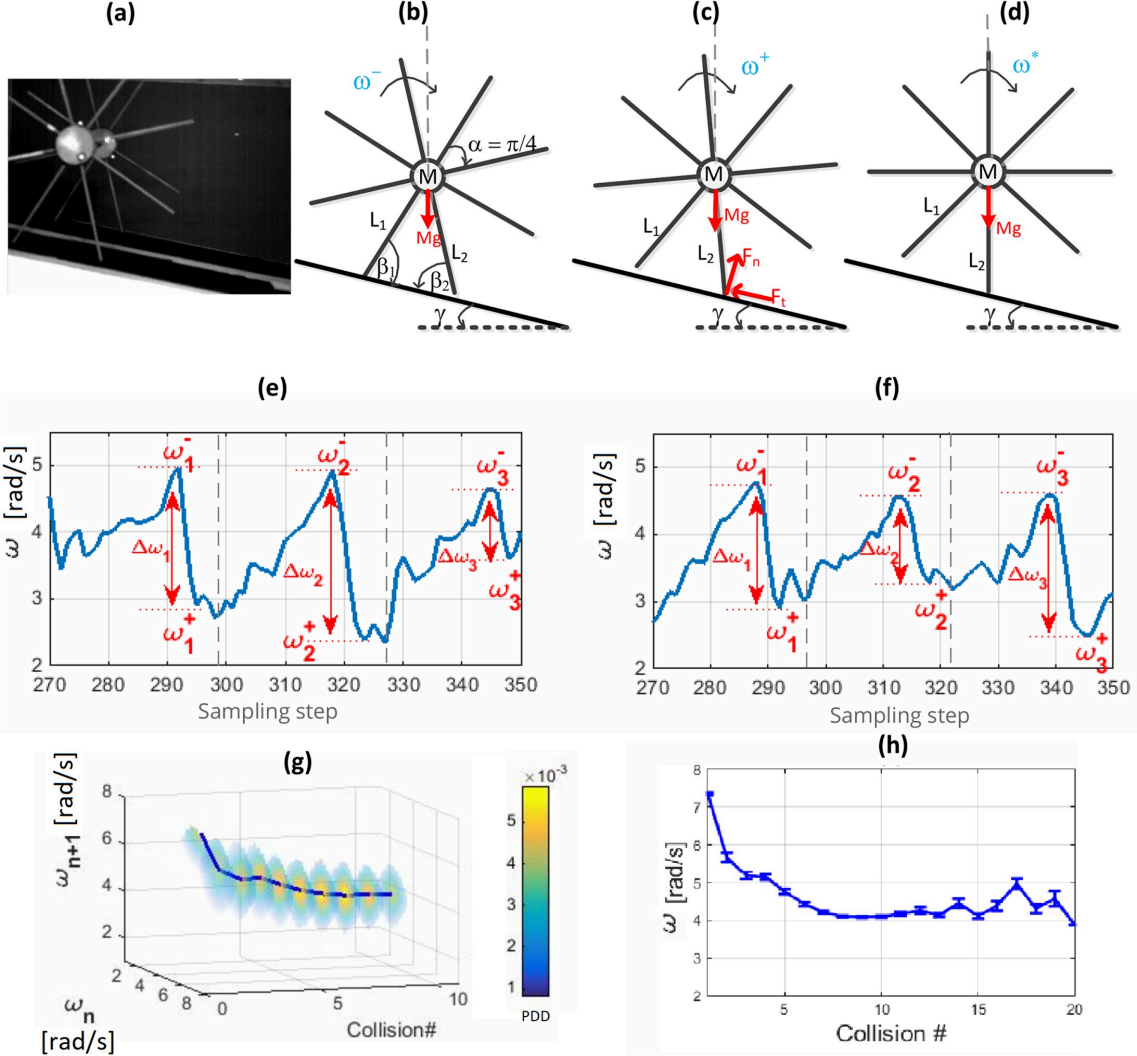
The motion of an 8 spoke RW as a meta-stable walker. **(a)** A snapshot of an example RW in passive motion on a slope. **(b)-(d)** Schematics of the side-view of the RW just before collision, just after collision and at the vertical position of leg L2. **(e), (f)** Continuous plots of the angular velocity during a few steps of two trials of an experimental setup of a RW. The angular velocities just before and just after collision are presented as *ω*^−^ and *ω*^+^ respectively and the angular velocity drop during collision is shown as Δ*ω*. The variability of Δ*ω* during each collision contributes to the state variability during the steady state. The vertical dashed lines represent the minimum angular velocity position per cycle corresponding to the mid-stance RW position shown in Fig 1(d). **(g)** Probability distribution of angular velocity just before collision (*ω*) in its return map space for 10 collisions for the 50 experiment trials. The color-bar shows the probability density distribution at each step showing a 3D Gaussian probability density distribution around the steady state at each step of the walk. **(h)** The mean and standard deviation of *ω* for experimental and simulated datasets of the RW.

The discrete dynamic model of a RW provides a classic example of steady state behavior of passive walkers and their meta-stability. Here, we discuss the simulated and experimental locomotion of a RW down a slope (which we also describe in [3],) and extend the discussion into analyzing the dynamics on the return-map space of angular velocity of the RW. The experimental setup is shown in Fig 1(a) and the forces acting on the RW is depicted in Fig 1(b) to 1(d) just before collision, just after collision, and with colliding leg vertical (i.e. the point where potential energy is highest and therefore has to be crossed in order for motion to continue).

The idealized RW model has several assumptions including point mass at hip, no slip, impulse collisions which are inelastic, and an instantaneous transfer of support between legs at collision. The authors in [3] discuss the validity of these assumptions and their contributions to the stochastic nature of the walk. They have also observed that when the systems state is expressed in terms of the angular velocity just before collision (*ω*), the steady state *ω*_*s*_ remained unchanged for a fixed *γ* (ramp slope) irrespective of the starting states [3]. Therefore, the state transitions of *ω* on its return map space behave like random walks initially biased towards the attractor at the steady state. This is also described in terms of the Poincaré section defined at each ground collision. The continuous state transitions of a dynamic walking systems are punctuated by ground collisions as shown earlier. These provide a natural time-discretization of the system onto a Poincaré map [9, 11, 18]. Poincaré maps provide the ability to study stability of nonlinear limit cycle behaviors in walking systems [2]. For the RW, the Poincaré sections for *ω* has been analyzed to draw important characteristics of the system [3, 10]. Although the local attractor at the meta-stable state is successful in overpowering most of the escape attempts, eventually the walker escapes from the attraction field of the steady state and reaches a global absorption state at failure. Our aim is to analyze the escape profile in terms of MFPT to escape from the meta-stable attraction field.

### Passive dynamic walking experiment for a RW

An experiment of a RW (same as that described in [3]) is fabricated out of a central aluminum body weighing 1.39 kg with 8 spokes connected at 45° angles (*α* = *π*/4) with an average leg length of 0.203 *m* from the center of the hub. The RW consisted of two parallel sets of 8-legged wheels rigidly coupled as shown in Fig 1(a) for static stability. Motion capture markers were attached to the central hub. A Vicon motion capture system was used to take 3D coordinates of markers at 120 frames per second. A 3.66 *m* long ramp was created out of standard construction timber and fixed to have a slope of 7.02°. Fifty trials of the experiment were carried out with near identical initial states achieved by releasing the wheel by the same leg from a steeper ramp of 30.59°. From the angular velocity graph, collision points were identified through a peak detection algorithm locating peak points that rise over 0.4 rad/s and separated by 45° angle segments (Fig 1(e) and 1(f)). The experimental data-set is attached as supplementary information in S1 Dataset.

We take the angular velocity (*ω*) at each point of collision as the state variable and use the return map space of *ω* as a continuous state space environment for the random walk of space-time motion of (*ω*,*t*). S1 Movie includes simulations of a few trials and how they map into random walks in state space. It was observed from Fig 1(g) and 1(h) that each walk is initially biased towards a steady/absorbing state around *ω*_*s*_ = 4.27 *rad*/*s* which acts as a local attractor (*ω*_*s*_ is obtained from averaging the last 50% of data for each trial). Even after reaching this field of attraction, variability was observed from Fig 1(g), where each slice shows the 3D Gaussian probability density distribution around the steady state at each collision. The probability of the *ω* at *n*^*th*^ collision being between any two arbitrary values can be obtained by integrating the probability density distribution (PDD) between those two *ω* values. The variance of each distribution determines the probability of exiting the attraction field of the meta-stable state and entering that of the failure state which would result in the walker coming to a stop.

### Variability in passive dynamic walking

The variability observed even after reaching a steady state attractor, as seen from Fig 1(g) and 1(h), characterizes metastability of a passive dynamic walker. This variability around the steady state attractor can push the system towards the absorbing failure state (0,0) given sufficient time.

Previous studies showed that the distribution of the coefficient of restitution and the coefficient of friction and their interactions play a dominant role in the steady-state variability of the RW [3]. Findings also show that other sources like the variability in leg lengths and starting speed do not play a significant role in steady-state variability.

Fig 1(e) and 1(f) presents the angular velocity (*ω*) profiles of the RW motion in two trials. The marked Δ*ω* at the collision demonstrates the intrinsic property of walking where the systems are punctuated by ground-foot collisions [3]. The variability of these ‘jumps’ result in the steady state variability discussed earlier. This in turn poses the risk of the walker reaching failure states also known as absorbing states. For the RW, we define the failure state as a state where *ω* = 0.

#### Simulating the RW’s motion down a ramp

The deterministic motion of the RW is discussed in detail in [3]. Following is a summary of this method which we use in this paper to simulate the above experiment to obtain a data-set consisting of 10,000 simulated RW runs.

The random walk of the state variable *ω* (angular velocity just before the collision with time on its return map space) is examined. A relationship of $\omega_{k}^{-}$ (the angular velocity measured just before *k*^th^ collision) to $\omega_{k+1}^{-}$ is used obtained in [3] using the conservation of energy during the collision. The total energy lost at collision (*ϵ*) is given in three components as the loss during the compression and restitution (*ϵ*_*n*_), energy lost in friction while slipping (*ϵ*_*t*_) and potential energy loss/gain when slipping up/down the ramp (*ϵ*_*pot*_). The momentum normal to the slope produces *ϵ*_*n*_ while that tangential produces *ϵ*_*t*_.

$$\begin{array}{c} \epsilon =\epsilon_{n}+\epsilon_{t}-\epsilon_{pot} \end{array}$$ (1)

where *ϵ*_*t*_ and *ϵ*_*n*_ are obtained by determining the energy lost during slip and slip-reversal separately as

$$\begin{array}{c} \epsilon_{t}=D_{pre}^{t}+D_{post}^{t} \end{array}$$ (2)

$$\begin{array}{c} \epsilon_{n}=D_{pre}^{n}+D_{post}^{n} \end{array}$$ (3)

where $D_{pre}^{t}$ denote the energy lost in the direction parallel to the slope during slip and $D_{post}^{t}$ is the same during slip reversal. Similarly, $D_{pre}^{n}$ and $D_{post}^{n}$ are energy lost normal to the slope. The potential energy gained due to slipping is obtained as

$$\begin{array}{c} \epsilon_{pot}=Mg\varsigma \sin\gamma \end{array}$$ (4)

where *M* is the central mass at hip, *ς* is the net/resultant slip in the direction of motion tangential to the ramp and *γ* is the slope. Then finally the relationship of *ω*_*k*+1_ to *ω*_*k*_ is obtained as

$$\begin{array}{c} {\left(\omega_{k+1}^{-}\right)}^{2}={\left(\omega_{k}^{-}\right)}^{2}+\psi \end{array}$$ (5)

where $\psi =\frac{2}{I}\left(2mgL\sin\gamma \cos\beta -\epsilon \right)$, *m* is the mass of the individual spoke of length *L*, *I* is the moment of inertia, $\beta =\sin^{-1}\{\left(L\sin\left(\alpha \right)\right)/\left(\sqrt[{}]{2}L\left(\sqrt[{}]{1-\cos(\alpha )}\right)\right\}$ where *α* is the angle between two spokes.

### Simulated motion summary

**Fig 2:**
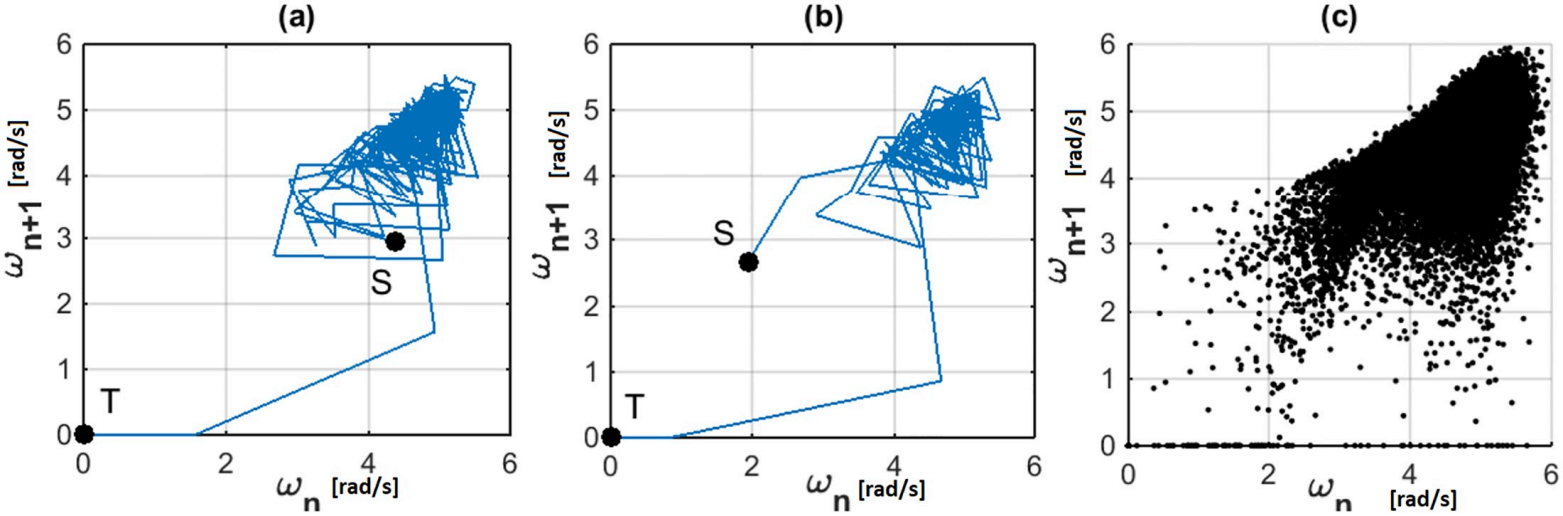
State transitions of the RW as random walks. **(a), (b)** Two sample trials of the simulated RW on Poincaré section corresponding to *ω*^−^. Initial (Source) and final (Target) states are shown as *S* and *T* respectively. **(c)** Ensemble of all state for 150 trials.

Fig 2(a) and 2(b) show two random walks for the RW system resulting from state transitions of *ω*. Fig 2(c) shows the simulated dataset of 150 trials. From these figures it is eminent that the simulted state transitions behave as anisotropic random walks. They are directionally biased by the local attractor formed by the metastable state transitions and the global attractor at the failure state.

**Fig 3:**
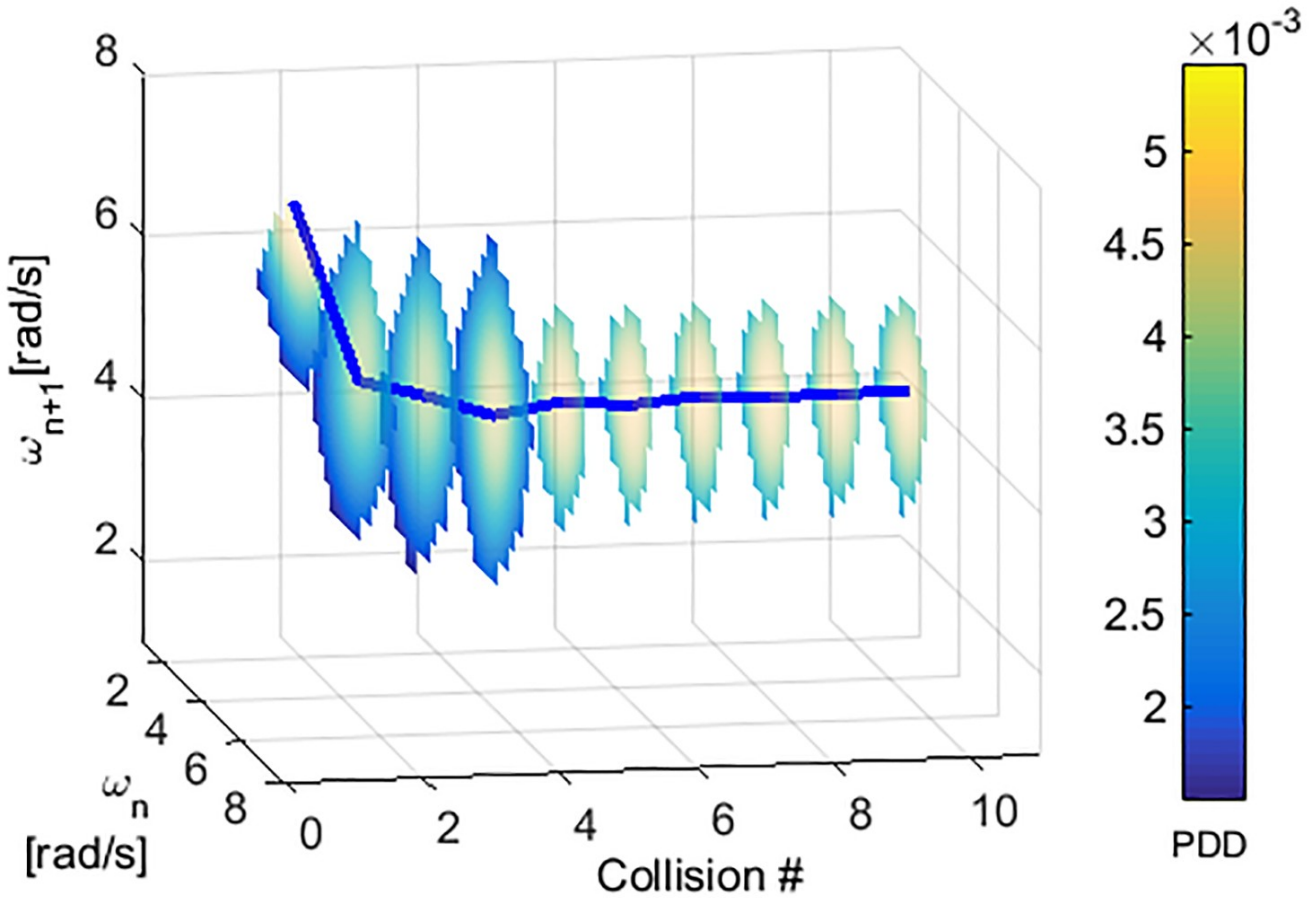
State distributions for first 10 collisions for a data-set of 10,000 simulations for Scenario I with $\mu =N(0.45,0.08^{2})$, $\eta =N(0.6,0.1^{2})$. The colorbar shows the probability density and each *i*^*th*^ slice shows the probability density distribution of (*ω*_*n*_, *ω*_*n*+1_) at the *i*^*th*^ collision.

A slice plot of the probability density distribution for the data-set of (*ω*_*n*_, *ω*_*n*+1_) for 10 collisions for a system with coefficient of friction at the contact point $\mu =N(0.45,0.08^{2})$ and coefficient of restitution $\eta =N(0.6,0.1^{2})$ is shown in Fig 3. Similar to the experimental results, it can be observed that even after reaching the steady state, there still exists slight variability for the state vector. From Fig 2(a) and 2(b) it is clear that when this variability moves the state vector outside the attraction filed of the steady state attractor (around (4.2,4.2) in this example), the state is attracted towards the failure state attractor (0,0)) which results in the walker coming to a stop. If the MFPT for the system state to reach a state outside the attraction field of the meta-stable attractor can be predicted, this knowledge can be used to improve the performance of the walker.

### MFPT prediction for state transitions modeled as random walks

General methods on MFPT estimation for random walks have advanced to finding efficient and accurate methods for walks on complex scale invariant media [17]. Such methods prove to be very efficient for a range of special networks including those on fractals [19], anomalous diffusion [17], scale-free networks [20, 21], bounded domains [22, 23], Lévy flights [17, 24], and Brownian motion [25]. But when the random walks are results of state transitions for real world dynamic systems, such ideal conditions are rarely met. These walks are anisotropic and commonly biased towards the attractors formed by non-linear interaction dynamics between the system and the environment [26]. In the following paragraphs we present a model which is able to predict the MFPT to reach arbitrary targets in biased media.

Given a state space of a random walk, two main transport properties are commonly used to describe random walks in terms of estimating MFPTs [17]. These properties represent the random walk with joint characteristics of the random walk (state transitions) as well as the state space. The first property is a measure of the reachability of nodes for the walker and expressed in terms of the density of nodes in the state space. Nodes are states the random walk can occupy and are networked by state transitions. A property known as the ‘fractal dimension’ (*df*) is used to quantify this and is commonly calculated using

$$\begin{array}{c} N\propto r^{df} \end{array}$$ (6)

where *df* gives the rate at which the number of nodes *N* increases with distance *r* from the source node [17]. In the case of a continuous state space, *df* follows the dimension of the state space. The other transport property important in estimating MFPT is the ease with which the walker can move away from the source. This is measured in terms of a ‘walk dimension’ (*dw*) which, for an unbiased network is calculated using

$$\begin{array}{c} t_{exit}\propto r^{dw} \end{array}$$ (7)

where *t*_*exit*_ is the exit time from a sphere of radius *r* from the source [27]. For a scale-free network this is the fractal dimension of the structure formed by the random walk. A biased network can be defined as one where there exists a concentration gradient in exit points from a sphere of a radius *r*. The effective bias modified walk dimension (*dw*_*b*_) is therefore direction dependent. The *dw* accounts for any obstacles or potential fields (on the state space) shaping the walk. The higher the value for *dw* is, the harder it is for the walker to leave its immediate neighborhood. For metastable systems *dw* is high resulting from the local attraction field of steady state.

The relative values for *dw* and *df* for a network give important insights for random walks on that network [28]. If *dw* < *df*, it signifies that the walk follows a ‘non-compact exploration’ meaning that the walker does not need to traverse a large part of the immediate neighborhood to exit from it. The significance of the source location is lost after a few steps for such random walks. In contrast, for the networks with *dw* ≥ *df* shows ‘compact-exploration’ where each node is eventually visited as obstacles make it slower for the walker to move away from the source. The authors in [17] show that for a length-scale-invariant network, the MFPT can be obtained as

$$\begin{array}{c} M\;F\;P\;T∼\{\begin{array}{cc} N(A-Br^{dw-df}) & \text{if}\;dw<df\\ N(A+B\ln r) & \text{if}\;dw=df\\ N(A+Br^{dw-df}) & \text{if}\;dw>df \end{array} \end{array}$$ (8)

where *N* is the number of nodes, ∼ indicates large *N* asymptotic equivalence and *A* & *B* are domain dependent constants. In this paper we propose that we can extend this method for networks showing directional bias, by bias-modifying the transport variables *df* & *dw*, and thereby using the MFPT knowledge to improve system performance. It is also worth noting that for a controller of a walker which shows isotropic state transitions, Eq (8) can be used as it is for MFPT predictions.

### Bias modified MFPT for minimal intervention control of passive dynamic walk of the rimless wheel

The objective of the proposed methods is to estimate the MFPT to reach any target node from any source node. Similar to most real world random walks like fire propagation given a wind direction, passive walking systems also show bias towards local and global attractors in their return maps. This is true for all metastable walking systems [9] where the metastable state transitions form local attractors and failure states form global attractors. We incorporated the effect of directional bias on the walker in the model with bias modification of transport variables.

In the existence of bias, these two transport variables need to be modified in order to account for the potential fields or obstacles shaping these walks [26, 29]. As a measure of the density of the nodes (i.e. set of states the random walk can occupy), and being only a property of the network (and not the walk), any existence of a bias on the network does not necessarily alter the calculation of *df*. Therefore, the fractal dimension in a biased network *df*_*b*_ can be calculated similar to the conventional approach. The exact methods of obtaining values for the variables *N* and *r* are dependent on the nature of the network and will be discussed further with a hypothetical set-up as well as using the example of the passive dynamics of a RW on a ramp later in the paper.

In contrast, the biased walk dimension *dw*_*b*_ should indeed account for any obstacles or potential fields shaping the walk. The general expression for *dw* cannot be applied to biased networks without modification. Firstly, the probability of reaching any given target point (*T*) is dependent on the relative angle between the line connecting the source (*S*) to *T* and the bias direction, and the intensity of bias. Therefore, with the increase of bias, although the rate at which the walker moves further away from *S* is higher, the angle segment in which the targets have a significant probability of reaching becomes smaller. Thus, the overall reachability of the network decreases with increasing bias intensity.

In order to obtain a generic method of calculating *MFPT* for biased walks, it was required to find a generic bias modified walk dimension *dw*_*b*_, for all nodes within a network primitive, such that the variances of *dw*_*b*_ and *df*_*b*_ are negligible for all nodes of the network.

### Obtaining bias modified *dw* in a generic network

**Fig 4:**
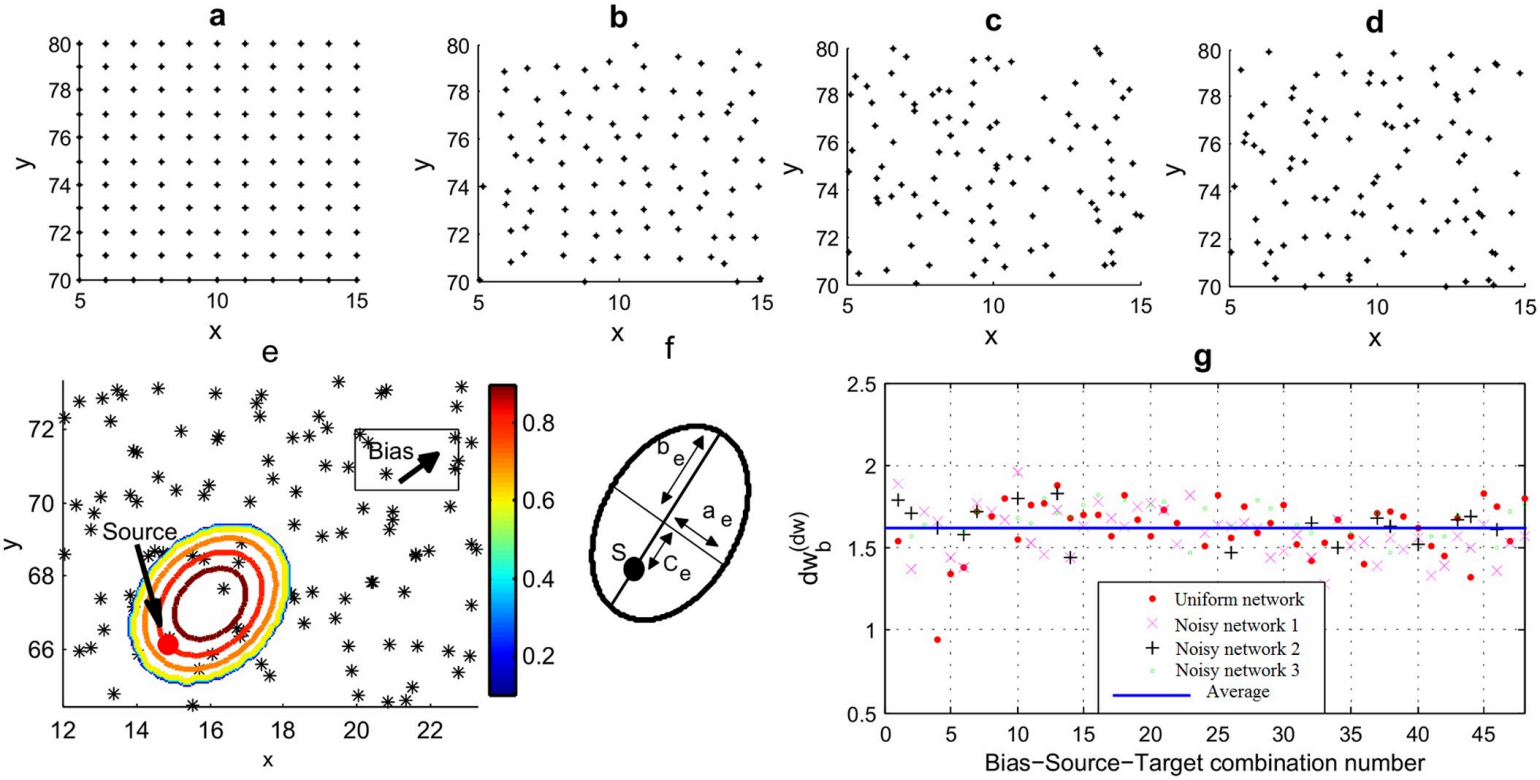
Obtaining a case invariant relationship between *dw* and *dw*_*b*_ for a 2D node distribution. Several hypothetical network primitives were considered including **(a)** a uniformly distributed network **(b)-(d)** and networks derived from the network in Fig 4(a) with added noise levels having standard deviations (*σ*) of 0.15, 0.5 and 0.8, respectively. **(e)** An example Gaussian *P*_*DD*_ (according to colorbar) for selecting next step in a random walk. Nodes shown as stars. **(f)** The dimensions of *P*_*DD*_’s base ellipse with major Eigen vector having the same direction as bias. (Refer S1 Text) **(g)** The relationship of *dw*_*b*_ to *dw* obtained empirically through comparing the values calculated for *dw* with the optimal *dw*_*b*_. The values for the four sample network scenarios in Fig 4(a)-(d) are shown with 48 Bias-Source-Target combinations each.

To explore the possibilities of empirically obtaining a generic *dw*_*b*_, we used a set of basic unconnected 2D node distributions. Starting with a collection of uniformly distributed node sets (*N* = 50 × 50) on 2D space (a section of which is shown in Fig 4(a)), the nodes were moved around by adding a Normally distributed noise with a fixed standard deviation (*σ*) for each case as

$$\begin{array}{c} \forall_{i}\left(x_{ni},y_{ni}\right)=\left(N\left(x_{i},\sigma \right),N\left(y_{i},\sigma \right)\right) \end{array}$$ (9)

where (*x*_*i*_, *y*_*i*_) are the coordinates of the *i*^th^ node without added noise (Fig 4(b)–4(d)). A network generated in this way becomes a ‘network primitive’ when the variance of transport properties at every node are negligible [29]. It is worth noting the importance of having the noise level fixed and low such that all nodes would still belong to the same primitive network. Networks with noise levels with *σ* ranging between 0-0.9 were considered in our simulations for the purpose of obtaining a case-invariant *dw*_*b*_. Each initialization of such network with *σ* > 0 is essentially different from the next even though both might share the same noise level. Therefore, several initializations were used for each noise level to assure the consistency of results.

Since like many network scenarios, the connectivity between nodes are dynamic [30, 31] and modified by the immediate neighborhood, the probability density distribution (*P*_*DD*_) of connectivity at each node is decided only once the walker reaches that node. Therefore, the next step was selected at each current step using a 3D Gaussian *P*_*DD*_ = *f*(*U*, *θ*, *R*) with elliptical contours oriented and shifted in the direction of the bias where *U* = bias intensity, *θ* = bias direction, and *R* = rate of spread. For each of these networks, we calculated the biased fractal dimension (*df*_*b*_) at each node using the count of nodes falling inside a circle of increasing radius *r* from each source node. The walk dimension (*dw*) was calculated using a Monte-Carlo approach by simulating a large number of random walks originating from a set of source nodes. Supplementary S1 Algorithm and S1 Text presents the calculation steps followed in obtaining the *dw* for each network scenario. This *P*_*DD*_ was inspired by a model that used Huygens principle to model random spreads [32] (a summary is presented in Supplementary S2 Text). The resulting *P*_*DD*_ for one step of a random walk is illustrated in Fig 4(e) and 4(f).

**Fig 5:**
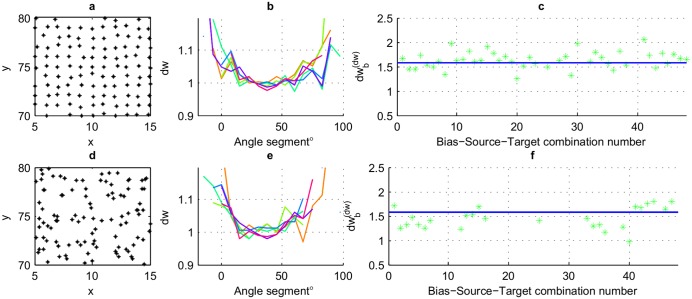
Obtaining *dw*_*b*_ from *dw*. **(a), (d)** Initializations of networks having noise levels of 0.1 and 0.7 respectively. **(b), (e)** Dependence of *dw* on the relative angle of the line connecting the source and target to the direction of bias for 6 initializations of networks with noise levels same as leftmost figures in each line. **(c), (f)** The relationship of *dw*_*b*_ to *dw* obtained empirically, for network on the left, through comparing the values calculated for *dw* obtained using Eq 7 with the optimal *dw*_*b*_ obtained using Eq 8. The values are taken from 48 Bias-Source-Target combinations for each network.

With the effect of bias, the ease of moving further from the source is dependent on the direction of the walk relative to bias. To capture this effect, the results were divided into angle segments when obtaining the *dw* for the hypothetical networks on 2D space using Monte-Carlo simulations. Fig 5(b) and 5(e) show the *dw* response for sample 2 systems with noise levels having *σ* = 0.1, 0.7 (Fig 5(a) & 5(d)) and a uniform bias towards a 30° angle. Six initializations of each noise level are considered in each graph. It can be seen that *dw* tends to be lowest in the direction of bias which is explained by the fact that the ease with which the walker moves further away from the source is highest in the direction of bias. However, the proposed method is able to predict MFPT for any direction using the corresponding *dw* values. The values for *dw* were obtained using curve fitting with non-liner least squares method for the simulated data-set of *t*_*exit*_ vs *r*.

Next we address the question of developing a generic method for obtaining *dw*_*b*_ for each scenario. A large number of network scenarios were considered in order to get a case invariant relationship between conventional *dw* with a bias modified *dw*_*b*_ [9 noise levels (*σ*) × 6 initialization × 8(*S*, *T*) sets × 6 different bias intensities].

In order to get deterministic *dw*_*b*_ values for each network scenario, the *MFPT* values were obtained numerically using Monte-Carlo simulations from a fixed source to reach a set of targets at different distances but in the same direction from the source. We considered each network as a small portion of a much larger domain and therefore a large value *L* (*L* = 10,000 for the simulations) was used as the *FPT* for instances where the target is never reached within the predefined maximum number of steps and the connection between the calculated MFPT* (*MFPT* taking *FPT* = *L* for misses) and *MFPT*^*r*^ (*MFPT* given the target is reached) is

$$\begin{array}{c} M\;F\;P\;T{\;}^{*}=\left(M\;F\;P\;T^{r}-L\right)P_{r}+L \end{array}$$ (10)

where *P*_*r*_ is the probability of reaching the target. This simulation was repeated for all 48 bias-source-target combination for each of the 9 network primitive types in 6 initializations each. The best curve fit for each scenario was obtained using curve fitting with the non-linear least squares method for the custom equation MFPT* = *N*(*A* − *Br*^*c*^) (following Eq (8)), with (*A*, *B*, *c*) being variables. From the achieved best fit, *dw*_*b*_ was obtained using *c* = *dw*_*b*_ − *df*_*b*_.

Addressing the question of deriving a generic *dw*_*b*_ from conventional *dw*, we compared the value sets of (*dw*, *dw*_*b*_) for over four hundred scenarios and for each a relationship was observed to follow an inverse power function of the form

$$\begin{array}{c} dw_{b}\propto C^{1/dw} \end{array}$$ (11)

where *C* is a constant.

**Fig 6:**
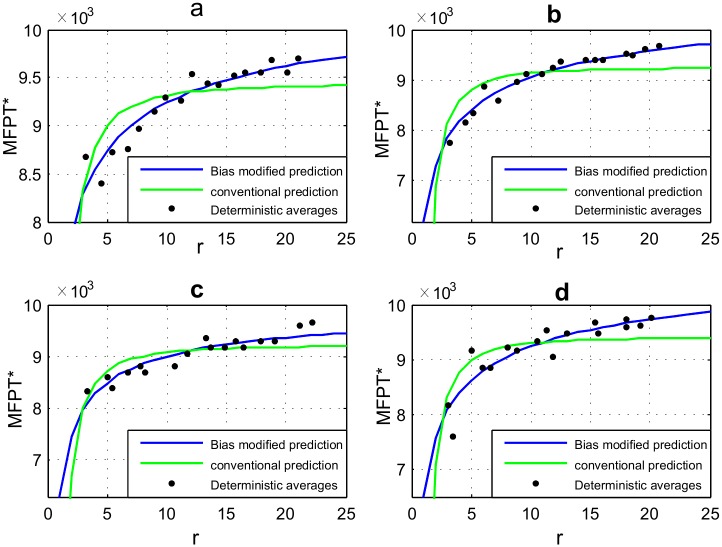
Numerically averaged MFPT*s using Monte-Carlo simulations (bold dots) compared to theoretical curve fits using bias-modified and conventional transport variables for networks with (a) (*U* = 1.2, *σ* = 0) (b) (*U* = 0.8, *σ* = 0.3), (c) (*U* = 1, *σ* = 0.5), (d) (*U* = 1.2, *σ* = 0.7). The r-squared value sets obtained for curve fitting using conventional and bias-modified variables for the four cases were [0.60713, 0.88098], [0.64693, 0.95868], [0.61473, 0.86012] and [0.65008, 0.82429] respectively.

The constant *C* was calculated empirically as being approximately *π*/2 for all tested scenarios (Figs 1(g), 5(c) and 5(f)) thus producing

$$\begin{array}{c} dw_{b}=(\frac{\pi }{2}{)}^{1/dw} \end{array}$$ (12)

as a generic relationship between *dw* and *dw*_*b*_. We show a few comparisons of MFPT estimates using conventional and bias modified transport variables in Fig 6. When compared to the deterministic counterparts (shown as dots), the figures make it evident how the modified transport variables facilitate a far superior estimation for *MFPT* for a random walk in a biased 2D network.

## Results

### Predicting the MFPT to failure for a PDW using the proposed method

**Fig 7:**
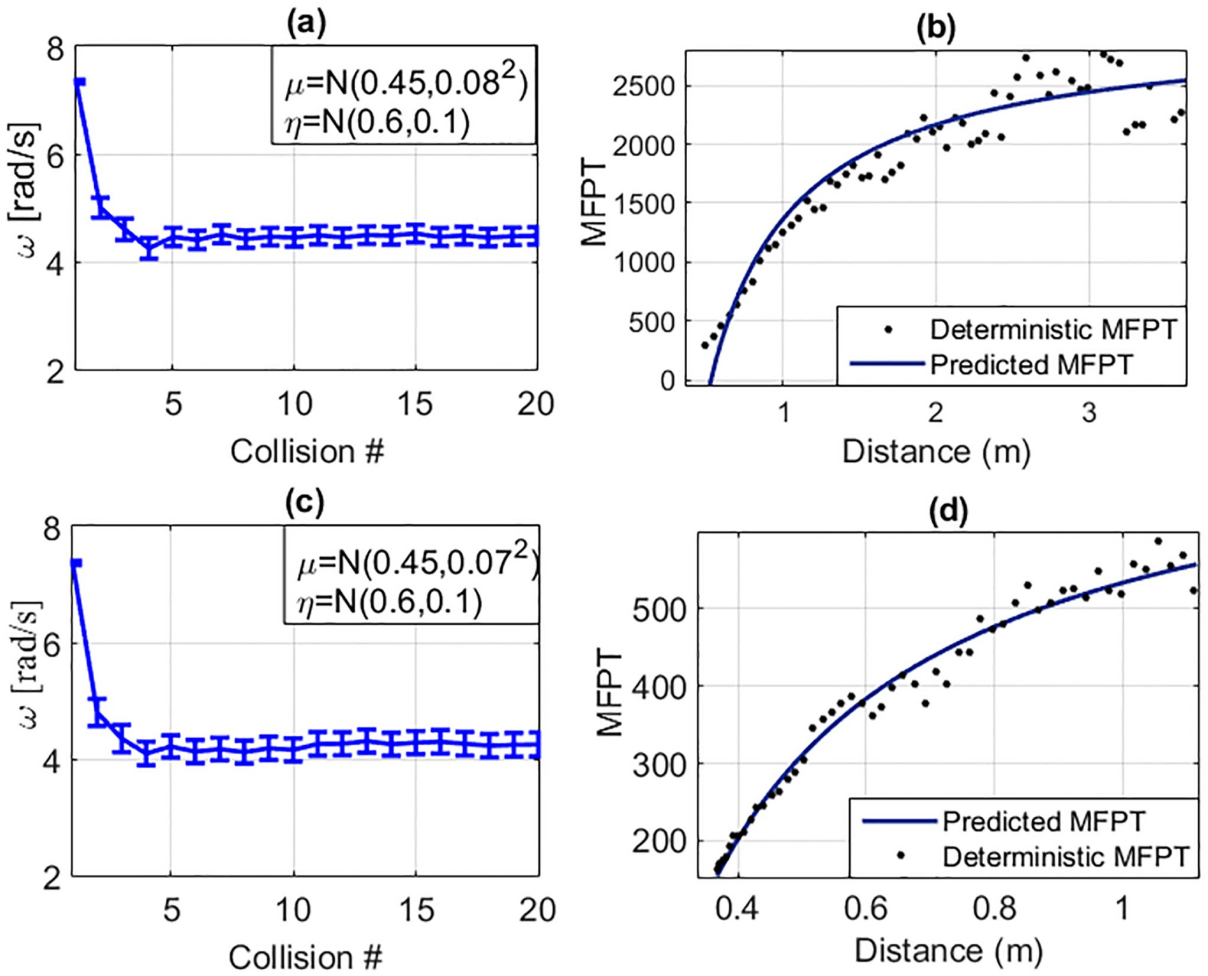
RW simulation **(a)** Scenario I: with $\mu =N(0.45,0.08^{2})$, η=N(0.6,0.1). The *ω* profile’s mean and standard deviation for the 10,000 simulations. **(b)** Comparison of the numerical MFPT (obtained through Monte-Carlo simulations) to the prediction. **(c)** Scenario II: with $\mu =N(0.45,0.07^{2})$, η=N(0.6,0.1). The *ω* profile’s mean and standard deviation for the 10,000 simulations. **(d)** Comparison of the numerical MFPT (obtained through Monte-Carlo simulations) to prediction.

**Fig 8:**
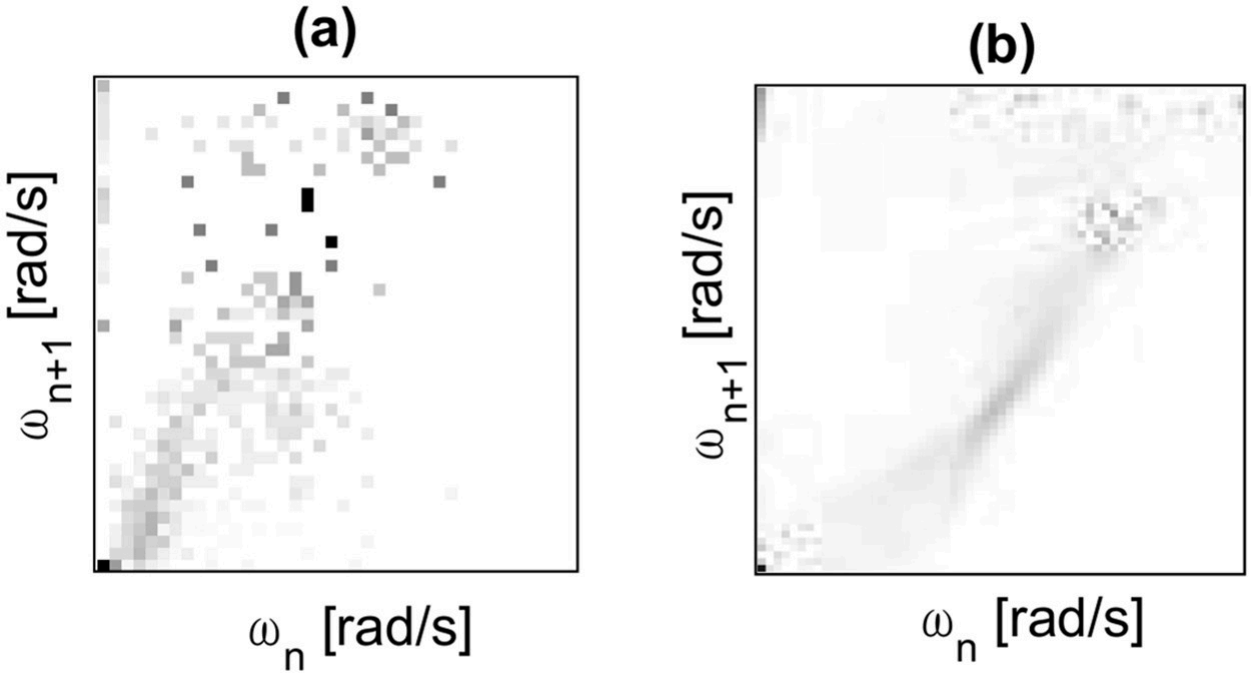
Transition probability matrices (TPMs) [9] obtained for (a) the experimental dataset of the motion of the RW down the slope and (b) for the simulated second data-set for Scenario I with $\mu =N(0.45,0.08^{2})$, $\eta =N(0.6,0.1^{2})$.

Here, we present the predictions of the MFPT to reach failure for the RW’s experimental data-set as well as data-sets for two simulated scenarios using the proposed method and the results compared with the actual numerical averages of the labelled data-set as well as predictions using the well-established method of using Transition Probability Matrices (TPMs). The authors in [9] present a model that predicts a global value for MFPT to reach failure, employing tools of stochastic process using a TPM for the system. A summary of the TPM method is included in Supplementary S5 Text. The results of the two simulated scenarios are depicted in Fig 7. The resulting TPM’s for the experimental data-set and one simulation scenario are included in Fig 8. Table 1 shows a comparison of these results. From Table 1, it can be observed that the proposed method produced predictions of MFPT to reach failure state comparable with the TPM method for the three presented scenarios. The accuracy of the proposed method was 10% higher with respect to the Markov chain based TPM method for the experimental data-set while prediction accuracy was on similar ranges for simulated scenarios.

**Table 1:** Predictions using the proposed method to calculate MFPT to failure compared with numerical first passage time (FPT) averages and predictions using the TPM method. Comparisons are presented for the experimental data-set and two simulated scenarios with [$\mu =N(0.45,0.08^{2})$ and $\eta =N(0.6,0.1^{2})$] and [$\mu =N(0.45,0.07^{2})$ and $\eta =N(0.6,0.1^{2})$] respectively.

Dataset	MFPT		
	Average FPT	TPM prediction	Proposed Prediction
**Experimental Dataset**	13.7	10.3	11.7
**Simulated scenario I**	603.6	635.9	594.9
**Simulated scenario II**	2334	2441	2533

### Minimal intervention control

Here, we demonstrate the significance of the proposed method by using the MFPT predictions as a feedback signal for a minimum intervention controller for increasing the time to failure for a RW. We use the return map (the map of rotary speed at (*k* + 1)^th^ step against that at *k*^th^ step) as the state-vector. We describe the properties of the network of nodes in this space (*k* − (*k* + 1) nodes) that can be visited by the RW using the MFPT prediction. We propose that by estimating the MFPT profile to reach the failure state, this strategy allows us to take timely action to postpone that failure instance to some estimated span of time in the future.

Conventional feedback controllers try to correct the state of the dynamic system at every sampling step. This approach can be viewed as a full intervention. However, as passive dynamic walkers have their natural steady state limit cycle attractors, no control intervention is needed at steady state except when the walker is about to reach failure states.

**Fig 9:**
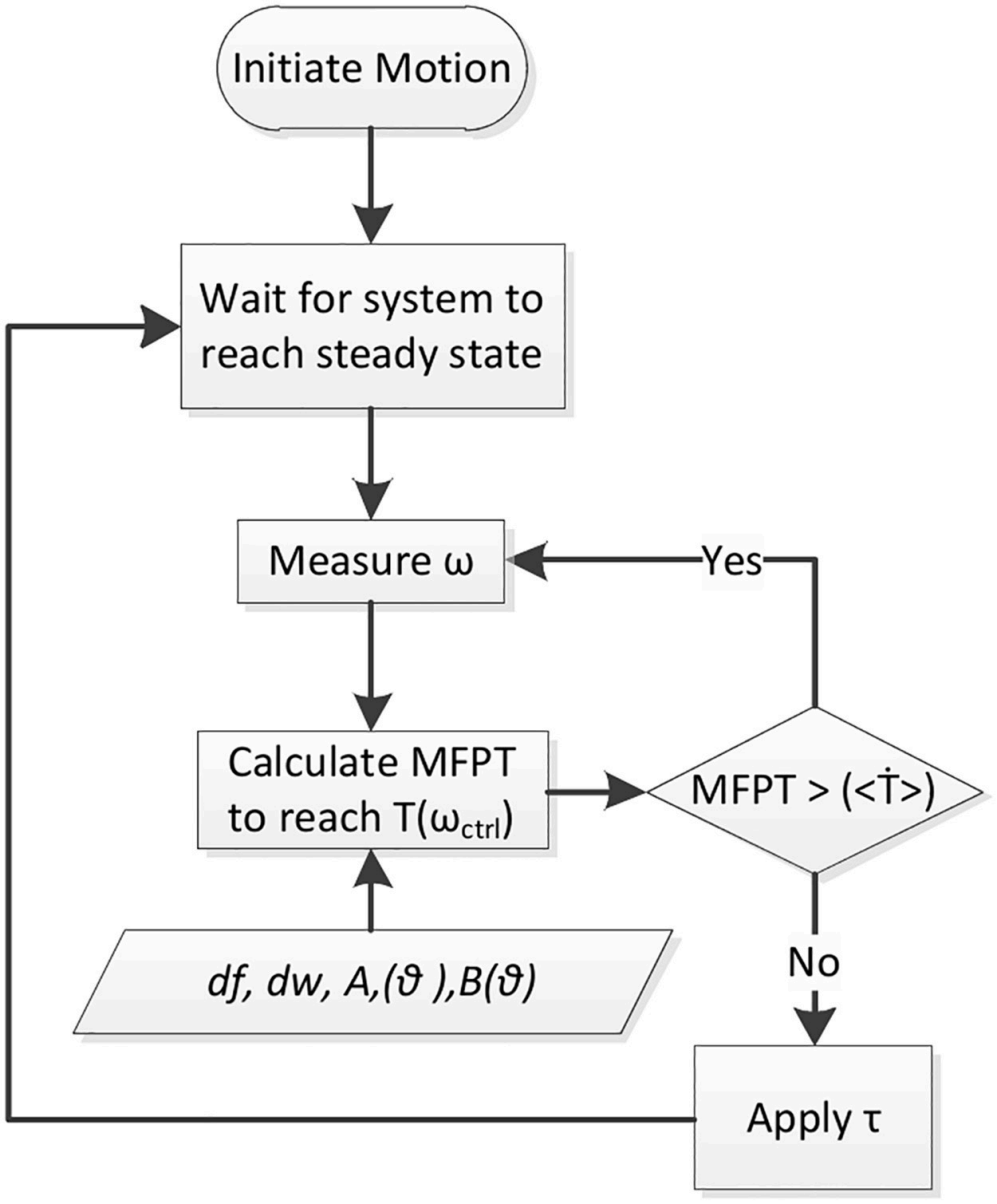
Flowchart of the MFPT based controller for the RW. Per step policy is used to decide if an external torque (*τ*) is applied depending on two control parameters; *T*(*ω*_*ctrl*_) which is the target point to which MFPT is calculated and $<\dot{T}>$ which is the threshold MFPT.

We propose by estimating the MFPT profile, a controller can allow the walker to stay in the steady state attractor filed, and take control action only when the MFPT drops below a threshold indicating imminent failure. We refer this to be a “minimal intervention” control strategy. The flowchart of the proposed minimal intervention controller is depicted in Fig 9 An external torque is injected “only” when required to avoid a failure state. Supplementary S2 Algorithm and discussion in supplementary S3 and S4 Text discuss the steps of the controller further.

The main advantage of the proposed MFPT prediction method is its ability to predict MFPT to reach ‘any’ target T in the state space. In the discussed controller we have manipulated this feature by using MFPT calculations to reach many intermediate states before reaching failure. This knowledge of the systems angular velocity profile as it reaches failure gives us more flexibility in selecting a better control policy through a better performing trigger point to inject an external torque (*τ*) into the system.

We calculate *dw*_*b*_ and *df*_*b*_ in the steady state region using the last 50% of data for each trial. The continuous two dimensional state space gives *df*_*b*_ = 2. The *dw*_*b*_ was calculated to be 1.9234 using *t*_*exit*_ ∝ *r*^*dw*^ and *dw*_*b*_ = (*π*/2)^(1/*dw*)^ for the data set of 50 experiment runs. From the case *df*_*b*_ > *dw*_*b*_, we compared the output of the proposed method using *MFPT* = *N*(*A* − *Br*^(*dw*_*b*_−*df*_*b*_)^), with the experimental data-set of the example of a RW on a ramp.

**Fig 10:**
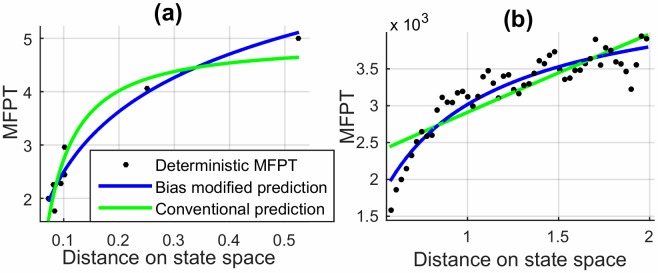
*MFPT* estimation for the angular velocity profile on state space for the motion of a passive RW on a ramp. **(a)** Step-wise *MFPT* comparison for experimental data and **(b)** for simulated data.

With the purpose of computing the mean number of steps to reach targets at different distances, a set of target states were selected with the walker initially at the steady state. For the 50 test cases, the times taken to reach these target states (within an allowed deviation) were calculated. Fig 10(a) compares the average time taken to reach targets of different distances from the steady state. The *MFPT*s obtained numerically from experimental data are shown as bold dots and compared with the theoretical curve fit to find *A* and *B*. A high goodness of fit for the training dataset was observed with an R-squared value of 0.9620 possible with the use of the *df*_*b*_ and *dw*_*b*_ values as proposed.

**Fig 11:**
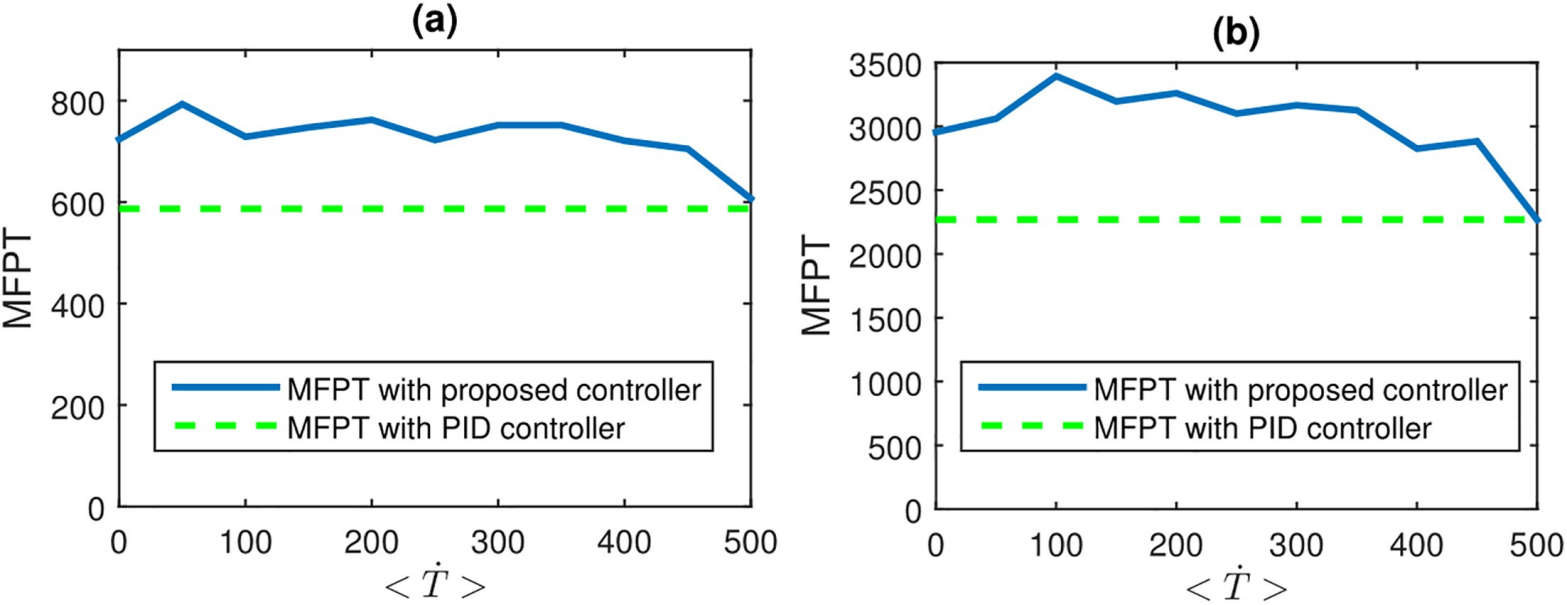
MFPT profile with respect to $<\dot{T}>$ compared to MFPT with the use of PID controller, both with *ω*_*ctrl*_ = 5*σ* for (a) scenario I ($\mu =N(0.45,0.08^{2})$ and $\eta =N(0.6,0.1^{2})$) and (b) scenario II ($\mu =N(0.45,0.07^{2})$ and $\eta =N(0.6,0.1^{2})$).

Fig 11 compares the MFPT profiles for the two presented scenarios with *ω*_*ctrl*_ = 5*σ* with the results of MFPT using a PID controller which tries to keep the *ω* above *ω*_*ctrl*_ at each collision. It was observed that the proposed controller was able to give 37.6% and 44.1% longer lifespans compared to the PID controller. In addition the PID controllers required 25.3% more applications of external torque for controlling. This reflects the energy efficiency of the proposed model.

## Discussion and conclusions

We have presented a novel method to compute the MFPT for a metastable walker to reach any known target state. This is then used in a minimal intervention controller that generates action only when the MFPT estimated from a given state falls below a threshold. Unlike Markov chain based approaches which give a global value for MFPT, the proposed method allows us to compute MFPT from any given state to any target state even if the target state is not an absorption state. Therefore, the proposed method can be used to evaluate alternative control policies or parameter changes of a given control policy by testing whether the new solution improves the MFPT or not. Moreover, the proposed method is able to predict accurately even when the state transitions show anisotropic probability density distributions. With the use of experimental and simulated datasets of a rimless wheel, we have demonstrated how metastable state transitions result in biased random walks in their state spaces.

Our results demonstrate high accuracy of MFPT predictions on a set of hypothetical random walks as well as simulated and experimental datasets of the passive motion of a rimless wheel. Comparisons with a TPM based method [9] demonstrate the accuracy of the model and its versatility in the flexibility provided by the option to have target states not limited to absorption states.

A control policy for a walking system needs to account for the stochastic aspects resulting from the variability induced by interactions with the surrounding environment. Reward based policies as such used in reinforcement learning literature are less effective for metastable systems. For example, a sequential policy is defined in [33] which generates movements for a robot, and which is optimized by a reward profile promoting behavior imitative of a human. Such methods become infeasible when the state transitions are metastable. In contrast, the proposed method uses a feedback signal of the state transitions for the system and the control policy does not require the system to learn a value function (a value function maps any state to an estimate of the total discounted sum of future rewards).

The basic requirement to use the proposed controller is to be able to map the state transitions of the system into a random walk in its state space. This is a common property for walking systems due to the inherent variability in interactions with the environment. The proposed minimal intervention control policy maps states into actions only when the MFPT falls below a threshold. A main advantage of this controller is that it can test any control policy anytime by evaluating them against the gain or loss of MFPT.

The main drawback in the proposed controller is that the accuracy depends on the dataset used to derive the transport variables. The next limitation is the need for the transport variables, *df* and *dw*, to be homogeneous throughout the state space. When the system has several state space attractors, the application of the proposed method is not straight forward. We have addressed this limitation using the concept of identifying ‘network primitives’ with a case study of cyclone prediction in [29].

Comparisons of the proposed minimum intervention controller showed significant improvements when compared to a PID based controller which tries to keep the angular velocity above a predefined threshold. An increase of up to 44.1% was seen for the lifespan of the walkers and the energy efficiency showed an increase of 25.3% with respect to using the PID based controller.

## Supporting information

S1 Text Calculating the walk dimension (dw) for the 2D network example. (PDF)

S2 Text A fire spread model using Huygens principle. (PDF)

S3 Text Control function for the RW using MFPT. (PDF)

S4 Text Predictive controller using MFPT estimation. (PDF)

S5 Text MFPT prediction using TPM. (PDF)

S1 Fig Predicting MFPT to reach steady state. Comparison for two scenarios.
(PDF)

S2 Fig Flowchart of the MFPT based controller for the RW. (PDF)

S3 Fig MFPT profile with respect to control threshold. (PDF)

S1 Algorithm Obtaining *dw* table for given source node. (PDF)

S2 Algorithm Control function using MFPT. (PDF)

S1 Movie Supplementary video. Biased random walks and the motion of a RW down a ramp.
(MP4)

S1 Dataset Supplementary data. Data of the RW experiment.
(MAT)
