# Supplementary material for: Predicting the mean first passage time (MFPT) to reach any state for a passive dynamic walker with steady state variability
Source: PLoS One. 2018 Nov 29;13(11):e0207665. doi: 10.1371/journal.pone.0207665 (PMC6264876; doi:10.1371/journal.pone.0207665)
Supplement: S1 Text — (PDF) [file pone.0207665.s001.pdf]

S1. Calculating the walk dimension (dw) for the 2D network example.

The steps followed to calculate the dw for a given source point (S) of a known dynamic 2D network is summarised in S1 Algorithm. The dw is calculated prior to the *MFPT* calculation and recorded in a matrix with respect to bias intensity ($bias\_range = 0.6 - 1.6$ for the simulation) and relative direction. The results are divided into angle segments ($angle\_segment\_range = -\pi : \pi/24 : \pi$ in the simulations) to capture the effect of bias on the ease with which the walker can move in different directions relative to bias. Therefore, the calculation is repeated for the possible range of bias intensities and iterated a large number of times ($max\_iterations = 500$ in the simulations) such that the average dw would converge into a stable value. Random walks are generated (Movie S1) having a predefined maximum number of hops per walk (max_hops). The status of each node is kept in a reference matrix (F), to capture the dynamicity of node availability and to identify obstacles. The next node at each hop is identified using a Probability density distribution (P_{DD}) which is a function of bias intensity (U), bias direction (θ) and the rate of spread (R) which is the speed of the walk without bias and is a property of the network. For the first example presented in the main text, the Length-to-Breadth ratio (LB), Head-to-Back ratio (HB), length of major axis (b_e), length of minor axis (a_e) and the shift of centre in the direction of bias (c_e) (Fig. ref(fig1)(b)) were obtained using

$$\begin{aligned} LB &= e^{0.25U} \\ HB &= LB^{2.5} \\ a_e &= 0.5 * (R + R/HB)/LB \\ b_e &= (R + R/HB)/2 \\ c_e &= b - R/HB \end{aligned} \tag{S1}$$

respectively. The equations for LB and HB are taken such that for an environment without bias, LB would be 1 and HB would be an augmentation of LB. The coefficients for LB and HB were selected at random. The orientation of the

ellipse is equal to θ. The Gaussian P_{DD} for selecting the next node is

$$P_{DD}(x, y) = exp(-(d_e(x - C_x)^2 + 2e_e(x - C_x)(y - C_y)+ \\ f_e(y - C_y)^2)) \tag{S2}$$

where (C_x, C_y) are the coordinates of the centre of the ellipse obtained by shifting the current position a distance of c in the θ direction. $d_e = cos^2\theta/2\sigma_x^2 + sin^2\theta/2\sigma_y^2$, $e_e = -sin2\theta/4\sigma_x^2 + sin2\theta/4\sigma_y^2$, $f_e = sin^2\theta/2\sigma_x^2 + cos^2\theta/2\sigma_y^2$ where σ_x and σ_y, the standard deviations in x and y directions, are chosen from a_e and b_e depending on θ. The next node is selected using P_{DD} and F. The distance and relative direction for the new node is calculated and recorded alongside the hop number (i.e. time-to-reach) with respect to the angle segment the new node belongs to. Finally dw is calculated using curve fitting ($dwcurvefit$) for time-to-reach(t_{exit}) versus distance(r) by getting the least square error.
