# Supplementary material for: Predicting the mean first passage time (MFPT) to reach any state for a passive dynamic walker with steady state variability
Source: PLoS One. 2018 Nov 29;13(11):e0207665. doi: 10.1371/journal.pone.0207665 (PMC6264876; doi:10.1371/journal.pone.0207665)
Supplement: S2 Text — (PDF) [file pone.0207665.s002.pdf]

S2. A fire spread model using Huygens principle

We chose our first example to be based on Huygens principle (HP) as it is generally known to be a universal model of propagation [1] for natural environments. It has been shown that HP applies to any propagation phenomena which can be described through explicit linear differential and difference equations.

The two-dimensional network with dynamic connectivity described in the main text is inspired by a two-dimensional deterministic fire growth model named FARSITE [2]. We use the section of FARSITE model that describes surface fire spread as the base for obtaining the probability density distribution (P_{DD}) for identifying the next hop of a random walk. The main approach is to apply HP at each vertex at the spread perimeter to shape and orient an elliptical wavelet at each time step. The size is determined by the spread rate and the length of a computation time step and the shape is determined by the direction and effective intensity of bias. The *LB* an *HB* for the FARSITE model are found using

$$\begin{aligned} LB &= 0.936e^{(0.2566U)} + 0.461e^{(-0.1548U)} - 0.397, \\ HB &= (LB + (LB^2 - 1)^{0.5})/(LB - (LB^2 - 1)^{0.5}) \end{aligned} \tag{S1}$$

formulated empirically [2]. In our example, we convert the wavelet into a probability density distribution, thus reducing the spread to a random walk.

[1] E. Peter, Huygens' principle and the modelling of propagation, European Journal of Physics 17 (4) (1996) 226.

[2] M. Finney, R. M. R. Station, FARSITE, Fire Area Simulator–model development and evaluation, US Department of Agriculture, Forest Service, Rocky Mountain Research Station, 1998.
