# Supplementary material for: Predicting the mean first passage time (MFPT) to reach any state for a passive dynamic walker with steady state variability
Source: PLoS One. 2018 Nov 29;13(11):e0207665. doi: 10.1371/journal.pone.0207665 (PMC6264876; doi:10.1371/journal.pone.0207665)
Supplement: S3 Text — (PDF) [file pone.0207665.s003.pdf]

S3. Control function for the RW using MFPT

The steps followed for the MFPT based control for the RW model is summarised in S2 Algorithm. A training dataset for the RW model under given conditions is used to obtain the transport variables $df_b, dw_b(\theta), A(\theta), B(\theta)$ for the network. For a continuous network, df_b follows the dimension of the network (n). The calculation for the other three variables were repeated for a set of angle segments θ_s. The mean number of collision to reach steady state (C_m) is either observed from the dataset or calculated with a separate MFPT estimation (similar to S1 Fig). The pre-identified MFPT threshold ($< \dot{T} >$) and control threshold (ω_{ctrl}) are then initialised along with the remaining variables. The rest of the steps are repeated at using the measure of ω_c at each collision. The MFPT to reach failure state ($< T >$) is calculated using the current state vector ($S_{\omega_c,\omega_{c-1}}$) and ω_{ctrl} and a torque (τ) is applied when $< \dot{T} >$ exceeds $< T >$.
