# Supplementary material for: Predicting the mean first passage time (MFPT) to reach any state for a passive dynamic walker with steady state variability
Source: PLoS One. 2018 Nov 29;13(11):e0207665. doi: 10.1371/journal.pone.0207665 (PMC6264876; doi:10.1371/journal.pone.0207665)
Supplement: S4 Text — (PDF) [file pone.0207665.s004.pdf]

S4. Predictive controller using *MFPT* estimation

In this section we present a simple closed-loop controller for the RW's motion using the proposed method for predicting MFPT. The main objective of the controller was to extend the motion of a passive RW by applying an external torque 'only' when the system approaches failure. The aim here is to utilize the natural attractor at the steady state to optimize the controller's effectiveness At this point τ is applied giving a net energy gain at collision as

$$\epsilon_{gain} = \epsilon_\tau + \epsilon_{pot} - \epsilon_n - \epsilon_t \tag{S1}$$

where ϵ_τ is the energy gained by the applied τ, ϵ_{pot} is the potential energy loss/gain when slipping up/down the ramp,ϵ_n is the energy loss during the compression and restitution and ϵ_t is the energy lost in friction while slipping. A few concerns were addressed in this model. Firstly, it has long been observed that external forces, if not properly timed and applied, tend to lower the stability for passive walkers rather than achieving the intended opposite. Therefore, it was crucial for the controller to have a general sense of the MFPT profile in real time to know the correct timing for external actuation. Secondly, it was important to keep the actuation minimal to preserve the energy budget which is a main goal of controlling an otherwise passive system. Finally, the controller needed to allow the system to converge to the steady state after each intervention.

The external torque applications are timed using the MFPT profile of the system to reach a given intermediate point (ω_{ctrl}) defined in terms of a multiple of σ distance away from failure state. Once the MFPT estimate to reach ω_{ctrl} goes below a predefined threshold $<\dot{T}>$, an external torque (τ) is applied. The steps of this controller are summarized in S2 Algorithm with a description in S5 text. The flowchart presented in S2 Fig describes the basic steps of the proposed closed-loop controller. We present the output of the controller in two simulations scenarios with $\mu = \mathcal{N}(0.45, 0.08^2)$, $\eta = \mathcal{N}(0.6, 0.1^2)$ and $\mu = \mathcal{N}(0.45, 0.07^2)$, $\eta = \mathcal{N}(0.6, 0.1^2)$ respectively. For each simulation scenario a training data-set

was used initially to get the transport variables ($df_b,\ dw_b(\theta),\ A(\theta),\ B(\theta)$) for a range of relative angle (θ) segments. These are kept in a look-up table for referencing in real-time control.

S3 fig shows a few comparisons of resulting MFPT profiles to reach a set of ω_{ctrl} settings calculated numerically after using the controller with an array of $< \dot{T} >$ values. It was observed that for the RW with conditions similar to simulation scenarios I and II, the best results were obtained when the controller was set to apply an external torque (τ) when the MFPT prediction goes below 50 steps and 100 steps respectively. Best results were observed for both presented scenarios with ω_{ctrl} set to 5σ. A 44.7% and 45.51% increase of life-span for the RW system was observed for the two scenarios respectively. It was also observed that it was possible for the performance to decrease if wrong timing was applied with the same control action.
