# Supplementary material for: Predicting the mean first passage time (MFPT) to reach any state for a passive dynamic walker with steady state variability
Source: PLoS One. 2018 Nov 29;13(11):e0207665. doi: 10.1371/journal.pone.0207665 (PMC6264876; doi:10.1371/journal.pone.0207665)
Supplement: S5 Text — (PDF) [file pone.0207665.s005.pdf]

S5. MFPT prediction using TPM

The global prediction of MFPT using the Transition Probability Matrix (TPM) in the main text follows the methods presented in [1] using metastable limit cycle analysis. The state distribution vector $\mathbf{p}[n]$ is defined as

$$\mathbf{p}[n+1] = \mathbf{p}[n]\mathbf{T} \tag{S1}$$

where $\mathbf{T}$ is the TPM such that

$$\mathbf{T}_{ij} = Pr(\mathbf{X}[n+1] = x_j | \mathbf{X}[n] = x_i) \tag{S2}$$

and each row of $\mathbf{T}$ sums to one. A special state x_1 is designated in $\mathbf{T}$ as an absorbing state representing all states of the walker at failure. Using eigenmode analysis and the characteristics of metastability, the authors in [1] show that the global/system MFPT for the can be predicted as

$$M \approx \frac{-1}{log(\lambda_2)} \approx \frac{1}{1-\lambda_2} \tag{S3}$$

where λ_2 is the second largest eigenvalue.

[1] K. Byl, R. Tedrake, Metastable walking on stochastically rough terrain, Proceedings of robotics: science and systems IV (2008) 6490–6495.
