# Supplementary material for: Predicting the mean first passage time (MFPT) to reach any state for a passive dynamic walker with steady state variability
Source: PLoS One. 2018 Nov 29;13(11):e0207665. doi: 10.1371/journal.pone.0207665 (PMC6264876; doi:10.1371/journal.pone.0207665)
Supplement: S1 Fig — Comparison for two scenarios. (PDF) [file pone.0207665.s006.pdf]

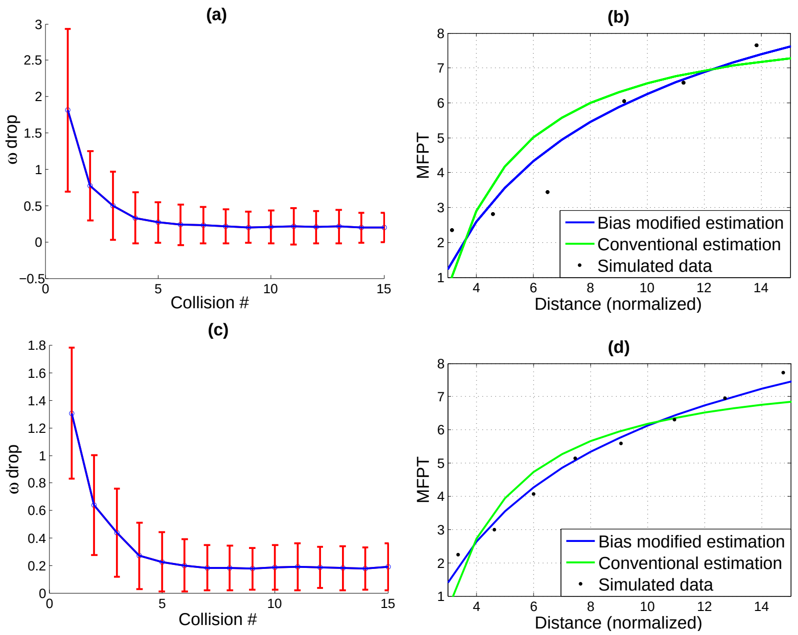

Figure S1: Prediction MFPT to reach steady state. **(a)** Scenario 1: Slope = $3.2°$, $\eta = 0.84$. The ω drop mean and standard deviation for the 1000 simulations. **(b)** Comparison of the numerical MFPT (obtained through Monte-Carlo simulations) to the theoretical estimations using conventional and bias modified transport variables. **(c)** Scenario 2: Slope = $5.2°$, $\eta = 0.54$. The ω drop mean and standard deviation for the 1000 simulations. **(d)** Comparison of the numerical MFPT (obtained through Monte-Carlo simulations) to the theoretical estimations using conventional and bias modified transport variables.
