# Supplementary figures and images for: Predicting the mean first passage time (MFPT) to reach any state for a passive dynamic walker with steady state variability
Source: PLoS One. 2018 Nov 29;13(11):e0207665. doi: 10.1371/journal.pone.0207665 (PMC6264876; doi:10.1371/journal.pone.0207665)

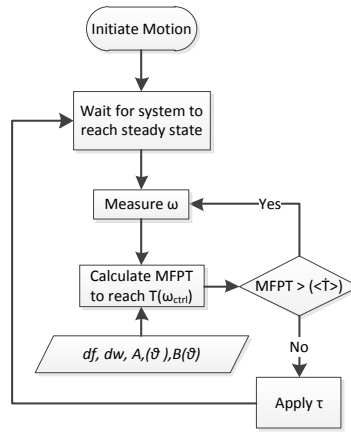

Figure S2: Flowchart of the MFPT based controller for the RW.

Supplement: S2 Fig — (PDF) [file pone.0207665.s007.pdf]
