# Supplementary material for: Predicting the mean first passage time (MFPT) to reach any state for a passive dynamic walker with steady state variability
Source: PLoS One. 2018 Nov 29;13(11):e0207665. doi: 10.1371/journal.pone.0207665 (PMC6264876; doi:10.1371/journal.pone.0207665)
Supplement: S3 Fig — (PDF) [file pone.0207665.s008.pdf]

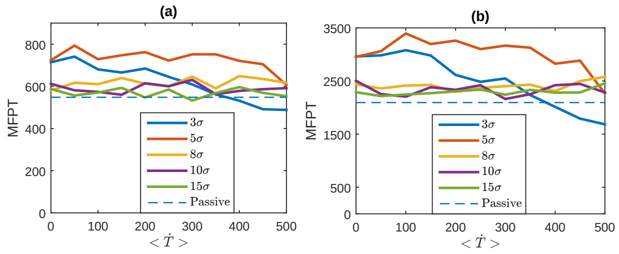

Figure S3: MFPT profile with respect to control threshold ($<\dot{T}>$) for several target points (ω_{ctrl}=3σ, 5σ, 8σ, 10σ, 15σ away from failure state defined as 2 [rad/s]) for **(a)** scenario I ($\mu = \mathcal{N}(0.45, 0.08^2)$ and $\eta = \mathcal{N}(0.6, 0.1^2)$) and **(b)** scenario II ($\mu = \mathcal{N}(0.45, 0.07^2)$ and $\eta = \mathcal{N}(0.6, 0.1^2)$). The dashed line gives the MFPT to failure for the passive system.
