# Supplementary material for: Predicting the mean first passage time (MFPT) to reach any state for a passive dynamic walker with steady state variability
Source: PLoS One. 2018 Nov 29;13(11):e0207665. doi: 10.1371/journal.pone.0207665 (PMC6264876; doi:10.1371/journal.pone.0207665)
Supplement: S1 Algorithm — (PDF) [file pone.0207665.s009.pdf]

Algorithm S1 Obtaining dw table for given source node

begin
  $[c_{sx}, c_{sy}]$ = coordinates of source node
  define $max\_iterations$, max_hops
  define max_hops
  initialise vectors $bias\_range$, $angle\_segment\_range$
for $\forall u \in \{bias\_range\}$ **do**
  for $i = 1 : max\_iterations$ **do**
    $obstacle\_reached = 0$
    for $s = 1 : max\_hops$ **do**
      coordinates of current node $\rightarrow [c_{1x}, c_{1y}] = \begin{cases} [c_{sx}, c_{sy}], & \text{if } s = 1. \\ [c_{2x}, c_{2y}], & \text{otherwise.} \end{cases}$
      define θ, $U \leftarrow f(u)$, R
      dynamic node status matrix $\rightarrow F$
      if $obstacle\_reached == 1$ **then**
        break;
      end if
Obtain P_{DD} for identifying next hop
      $P_{DD} \leftarrow f(c_{1x}, c_{1y}, \theta, U, R)$
      coordinates of next node $[c_{2x}, c_{2y}] \leftarrow f(P_{DD}, F)$
      if $[c_{2x}, c_{2y}]$==null **then**
        $obstacle\_reached = 1$
        continue
      end if
      $dist = \sqrt{((c_{2x} - c_{1x})^2 + (c_{2y} - c_{1y})^2)}$ Find angle segment the node belongs to
      $hop\_dir = \pm tan^{-1}((c_{2y} - c_{1y})/(c_{2x} - c_{1x}))$
      $hop\_segment \leftarrow f(angle\_segment\_range, hop\_dir)$
Update vectors
      if $max(t_{exit}(u, hop\_segment, :)) < dist$ **then**
        $t_{exit}(u, hop\_segment, end + 1) = s$
        $r(u, hop\_segment, end + 1) = dist$
      end if
    end for
  end for
Update table
  for $\forall seg \in \{angle\_segment\_range\}$ **do**
    $dw(S, u, seg) = dw_n \leftarrow dwcurvefit(t_{exit}, r; t_{exit} = a \times r^{dw_n})$
  end for
end for
