# Supplementary material for: Predicting the mean first passage time (MFPT) to reach any state for a passive dynamic walker with steady state variability
Source: PLoS One. 2018 Nov 29;13(11):e0207665. doi: 10.1371/journal.pone.0207665 (PMC6264876; doi:10.1371/journal.pone.0207665)
Supplement: S2 Algorithm — (PDF) [file pone.0207665.s010.pdf]

**Algorithm S2** : Control function using MFPT

**Evaluate**
**if** continuous state space **then** $df_b \leftarrow n$
**else**
  $df_b \leftarrow N \propto r^{df_b}$
**end if**
**for** Angle segment $\theta_s \in \{\theta_1, \ldots, \theta_m\}$ **do**
  $dw(\theta_s) \leftarrow t_{exit} \propto r^{dw}$ & $dw_b = (\pi/2)^{1/dw}$
  $A(\theta_s), B(\theta_s) \leftarrow < T >= N(A(\theta_s) - B(\theta_s) r^{(dw_b(\theta_s) - df_b})$
**end for**
Mean # collisions to reach steady state = $C_m$
Control threshold = $\omega_{ctrl}$
Time threshold = $< \dot{T} >$
Step counter for steady state = $C_c$
$C_c = C_m$
**for** collision $c \in \{c_2, \ldots, c_m\}$ **do**
  Torque $\tau = 0$
  Measure angular velocity= $\omega_c$
  New state= $S_{\omega_c, \omega_{c-1}}$
  **if** $c == c_2$ **then**
    $C_c = C_c - 1$
  **else if** $((< T >) < (< \dot{T} >) \& (C_c < 0)$ **then**
    Apply torque $\tau = \tau_c$
    $C_c = C_m$
  **else**
    $C_c = C_c - 1$
  **end if**
  remove torque $\tau = 0$
  $r = distance((\omega_{ci}, \omega_{c(i-1)}(, (\omega_{ctrl}, \omega_{ctrl}))$
  $\phi = \pm tan^{-1}((\omega_{ctrl} - \omega_{ci},)/(\omega_{ctrl} - \omega_{c(i-1)}))$
  $< T >= N(A(\phi) - B(\phi) r^{(dw_b(\phi) - df_b)})$
**end for**
